# Using latent class analysis to characterize a high-misconception profile for targeted health education among HBsAg-positive pregnant women in China

**DOI:** 10.3389/fpubh.2026.1817867

**Published:** 2026-06-23

**Authors:** Gaiyan Du, Ping Zhang, Qiaojun Liu

**Affiliations:** 1 Public Health Department, Shanxi Bethune Hospital, Shanxi Academy of Medical Sciences, Tongji Shanxi Hospital, Third Hospital of Shanxi Medical University, Taiyuan, Shanxi, China; 2 Taiyuan Center for Disease Control and Prevention, Taiyuan, China; 3 Health Education and Promotion Department, Taiyuan Center for Disease Control and Prevention, Taiyuan, China

**Keywords:** health literacy, health misconceptions, hepatitis B virus, latent class analysis, mother-to-child transmission, pregnant women

## Abstract

**Background:**

Despite effective interventions, mother-to-child transmission (MTCT) of hepatitis B virus (HBV) remains a concern. While health education is crucial, knowledge gaps and misconceptions persist among HBsAg-positive pregnant women. Traditional variable-centered approaches often treat this population as homogeneous, potentially overlooking distinct cognitive subgroups that require tailored interventions. This study aimed to apply person-centered latent class analysis (LCA) to identify and characterize such latent cognitive profiles regarding HBV transmission knowledge.

**Methods:**

We conducted a secondary LCA on cross-sectional survey data from 203 HBsAg-positive pregnant women in Taiyuan (2020–2022). Model fit was assessed using BIC and entropy. Derived classes were characterized and compared using Knowledge and Misconception scores (Wilcoxon test) and characterized demographically and clinically.

**Results:**

LCA identified two profiles: a “Low-Misconception & High-Knowledge” profile (90.2%) and “High-Misconception & High-Knowledge” profile (9.8%) —a group demonstrating accurate knowledge of true transmission routes alongside persistent endorsement of casual transmission myths. The latter group endorsed numerous casual transmission myths (e.g., via food) despite knowing true routes, with a significantly higher Misconception Score. This group was younger, less reliant on internet information, and higher education was a protective factor against belonging to it.

**Conclusion:**

Using LCA, we identified a significant minority profile characterized by the co-occurrence of accurate knowledge and a high burden of casual transmission myths. This demonstrates the critical need for a precision public health approach to move beyond one-size-fits-all education. Screening for this cognitively conflicted subgroup and delivering myth-debunking counseling through trusted channels can optimize MTCT prevention efforts. Our findings provide an empirical basis for developing brief clinical screening tools and implementing tailored patient education during routine antenatal visits.

## Graphical abstract

**Figure:**
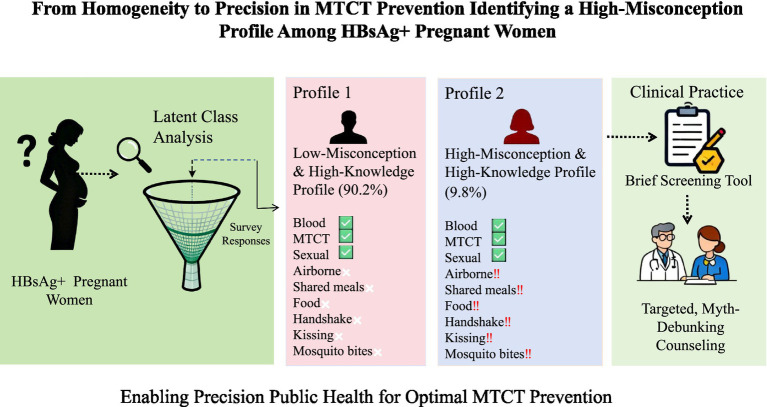

## Introduction

Hepatitis B virus (HBV) infection remains a formidable global public health challenge, with mother-to-child transmission (MTCT) constituting a primary pathway to chronic infection (1, 2). Despite the availability of highly effective prophylactic measures, including universal neonatal vaccination and antiviral therapy for pregnant women with high viral loads, breakthrough infections persist (3, 4). The success of these biomedical interventions is inextricably linked to maternal awareness, understanding, and adherence. Consequently, health education aimed at improving HBV-related knowledge is a cornerstone of comprehensive MTCT prevention strategies (5, 6).

China has made remarkable strides in reducing HBV prevalence through decades of sustained vaccination efforts (7). The integration of universal, free antenatal HBV screening into the national prevention of MTCT program since 2015 represents a pivotal policy advancement, successfully identifying a large number of HBsAg-positive pregnant women, many of whom are newly diagnosed. However, significant knowledge gaps and persistent misconceptions about transmission routes among this key population continue to be documented, posing a barrier to optimal engagement in the prevention cascade (6, 8).

However, the effectiveness of this policy is mediated by the complex socio-cultural landscape in which pregnant women navigate their health. Newly diagnosed women, in particular, must rapidly process technical medical information within a context potentially fraught with misinformation, stigma, and evolving family dynamics. Their understanding of transmission risks is not formed in a vacuum but is shaped by their access to and trust in various information sources (digital vs. clinical), educational background, and prevailing societal attitudes toward infectious diseases. Thus, a nuanced investigation into the heterogeneous cognitive profiles within this population is not merely an academic exercise but a necessary step to ensure that policy advances translate into empowered, informed, and stigma-free pregnancy experiences.

However, a critical limitation persists. Most existing research relies on variable-centered approaches (e.g., comparing mean knowledge scores), which implicitly assumes HBsAg-positive pregnant women are a homogeneous group in terms of health cognition (9). This approach may obscure meaningful within-population heterogeneity, distinct subgroups with qualitatively different belief patterns that may necessitate different intervention strategies (10). Person-centered analytical methods, such as latent class analysis (LCA), provide a powerful alternative by identifying unobserved (latent) subgroups based on individuals’ response patterns (11–13). Applying LCA to HBV knowledge can shift the research question from ‘what is the average knowledge level?’ to ‘are there distinct cognitive profiles, and what characterizes a high-risk profile?’ This study aims to fill this gap by employing LCA to empirically identify and characterize latent cognitive profiles, with a focus on uncovering a high-misconception subgroup that represents a tangible target for precision health education.

Applying LCA to the domain of HBV knowledge among pregnant women can address a salient research gap: moving beyond asking “what is the average knowledge level?” to ask “are there distinct cognitive profiles, and if so, what characterizes them?” Identifying a potentially high-risk profile characterized by a significantly higher burden of misconceptions alongside a distinct set of sociodemographic and behavioral features, which may support targeted, actionable interventions, would provide a nuanced, actionable understanding of vulnerability (14, 15).

To address this gap, a person-centered analytical approach is needed to uncover whether meaningful, qualitatively distinct cognitive profiles exist within this seemingly homogeneous population. Therefore, this study aimed to employ LCA to: (1) identify latent cognitive profiles regarding HBV transmission knowledge among HBsAg-positive pregnant women in China, and (2) comprehensively characterize the demographic, clinical, perceptual, and behavioral features of the derived profiles, with a particular focus on identifying a high-risk subgroup for targeted health education. Notably, we hypothesized that such a subgroup might exhibit a pattern of “cognitive conflict” – accurately endorsing true transmission routes while simultaneously endorsing common misconceptions – a phenomenon that standard knowledge scales would obscure by averaging correct and incorrect responses into a single score.

## Materials and methods

### Study design and setting

This secondary analysis utilized data from a hospital-based, cross-sectional study conducted in Taiyuan, Shanxi Province, China, between March 2020 and October 2022. The primary study aimed to assess hepatitis B virus (HBV)-related knowledge and health education needs among HBsAg-positive pregnant women, and its detailed methodology has been previously published (16). The current analysis applies latent class analysis (LCA) to the same dataset to identify distinct, empirically derived subgroups based on patterns of HBV transmission knowledge.

### Study population and sampling

The study population consisted of pregnant women with documented HBsAg positivity, recruited from five tertiary obstetric hospitals in Taiyuan. Inclusion criteria were: (1) documented HBsAg positivity in medical records; (2) singleton pregnancy; (3) gestational age of at least 12 weeks; (4) ability to understand and complete the questionnaire independently; and (5) provision of written informed consent. Exclusion criteria were: (1) coinfection with HIV, HCV, or other severe comorbidities such as decompensated cirrhosis; (2) cognitive impairment or psychiatric disorders that could interfere with participation; (3) refusal to participate.

From an initial sampling frame of 412 eligible women identified through routine prenatal screening, a final sample of 203 participants was obtained using a list-based simple random sampling method to enhance representativeness and reduce selection bias, yielding a response rate of 49.3%. The total sample size (*N* = 203) is generally considered sufficient for exploratory latent class analysis, which can provide stable parameter estimates for the overall model (12). However, we acknowledge that if a small latent class is identified, its sample size may fall below the ideal recommendation of 50–100 per class, which would primarily affect the statistical power for subsequent between-group comparisons and characterization, rather than the model’s ability to detect the presence of such a class. This sample size also satisfied the requirements of the parent cross-sectional study, which was calculated based on an estimated proportion of 0.5, a 95% confidence level, and a 7% margin of error.

### Data collection and measures

Data were collected via a structured, self-administered questionnaire hosted on the Wenjuanxing platform, as described in the primary publication (16). The questionnaire, which demonstrated acceptable internal consistency (Cronbach’s alpha = 0.78) (16), covered sociodemographic characteristics, HBV-related knowledge, perceptions, and information sources. For the current LCA, the analysis focused specifically on participants’ knowledge of HBV transmission routes. Nine binary (Yes/No) items assessed beliefs about both correct and incorrect transmission modes:

Correct transmission routes: Blood transmission, Mother-to-child transmission, Sexual transmission.

Common misconceptions (incorrect routes): Airborne transmission, Food transmission, Shaking hands with patients, Kissing, Eating at the same table, Mosquito/insect bites.

Responses were coded as 1 for a correct belief (i.e., affirming a true route or rejecting a false route) and 0 for an incorrect belief.

### Coding and score calculation

For the LCA modeling, all nine binary items were coded such that a response indicating a correct belief was coded as 1, and an incorrect belief was coded as 0. A correct belief was defined as: affirming a true transmission route (for the three correct routes) or rejecting a false transmission route (for the six misconception items).

For the purpose of creating external validation scores (the Knowledge Score and Misconception Score) that are intuitively easy to interpret, the coding for the six misconception items was reversed before summation. Specifically, for these six items only, an endorsement of the misconception (i.e., an incorrect belief) was assigned a value of 1, and a rejection of the misconception (i.e., a correct belief) was assigned a value of 0.

Consequently:

Knowledge Score (range 0–3): Sum of correct responses to the three true transmission routes. Higher score indicates better knowledge.
Misconception Score (range 0–6): Sum of (reversed) endorsements of the six non-transmission routes. A higher score indicates a greater number of held misconceptions.

### Statistical analysis

All analyses were performed using R software (version 4.3.1). Descriptive statistics summarized the sample characteristics. The parent study employed Pearson’s chi-square tests, multiple linear regression, and logistic regression to analyze knowledge scores and associated factors.

The core analysis of the current study involved Latent Class Analysis (LCA), a person-centered statistical method used to identify unobserved (latent) subgroups within a population based on their response patterns to observed categorical variables.

LCA model specification: The nine binary knowledge items were specified as manifest indicators. We fitted a series of latent class models, ranging from 1 to 4 classes, using the poLCA package.

Model selection: The optimal number of classes was determined by evaluating multiple model fit indices, including the Akaike Information Criterion (AIC), Bayesian Information Criterion (BIC), sample-size adjusted BIC (SABIC), and entropy (a measure of classification certainty, ranging from 0 to 1, with values >0.8 indicating clear separation), in addition to clinical interpretability and parsimony. The selected model exhibited high classification accuracy.

Validation of latent classes: To provide an interpretable and clinically meaningful characterization of the derived classes, we compared them on two summary scores derived from the LCA indicators. While these scores are not independent of the LCA model estimation, their construction differs from the binary coding used for LCA (see Coding and Score Calculation). Specifically, we calculated a Knowledge Score (sum of correct responses to the three true transmission routes, range 0–3) and a Misconception Score (sum of [reversed] endorsements of the six non-transmission routes, range 0–6; see Coding and Score Calculation in Methods). These scores offer a direct, intuitive quantification of each class’s level of factual knowledge and burden of misconceptions. Differences between classes on these continuous scores were tested using the non-parametric Wilcoxon rank-sum test, appropriate for the non-normal distribution of the scores.

Characterization of latent classes: Once the final LCA model was selected and classes were labeled thematically, we characterized the demographic, clinical, and perceptual profiles of each class. Categorical variables were presented as counts and percentages, and continuous variables as means and standard deviations or medians and interquartile ranges as appropriate. Group comparisons for categorical variables were conducted using Fisher’s exact test due to the small sample size in one identified class.

Sample size consideration for regression analysis: Given the small sample size of the cognitive conflict profile (*n* = 19), conducting a reliable multivariable regression analysis was precluded due to insufficient statistical power according to the “events per variable” criterion (17). Therefore, only univariable logistic regression was performed to explore factors associated with class membership, with the understanding that these findings require validation in larger samples.

### Ethics approval and consent to participate

The study was approved by the Scientific Research Ethics Review Committee of the Taiyuan Center for Disease Control and Prevention (Approval No. 2020001; approved on October 15, 2020). Prior to participation, written informed consent was obtained from all individual participants. The study adhered to the principles of the Declaration of Helsinki and followed national guidelines for biomedical research involving human participants.

## Results

A total of 203 pregnant women with HBV were enrolled and all provided complete responses on the nine HBV transmission belief items, therefore all 203 participants were included in the final Latent Class Analysis (LCA).

### Model selection and fit indices

The fit indices for the competing 1- to 4-class LCA models are presented in Table 1. The 2-class model demonstrated the lowest BIC (1573.62) and SABIC (1513.42) values, alongside an exceptionally high entropy (0.969), indicating superior model fit and excellent classification certainty. Although the 3- and 4-class models had marginally lower AIC values, their BIC and SABIC were higher, and their entropy values (0.758 and 0.804, respectively) were below the recommended threshold of 0.8. Therefore, the 2-class solution was retained as optimal based on statistical fit, parsimony, and interpretability.

**Table 1:** Fit indices for the 1- to 4-class latent class analysis models (*n* = 203).

Number of classes	AIC	BIC	SABIC	Entropy	Smallest class size (*n*)	Smallest class proportion (%)
1	1613.39	1643.21	1614.70	NA	203	100.0
2	1510.66	1573.62	1513.42	0.969	20	9.8
3	1487.86	1583.94	1492.06	0.758	17	8.4
4	1476.33	1605.55	1481.98	0.804	15	7.4

### Latent class analysis of HBV transmission knowledge

The LCA model identified two distinct latent classes based on participants’ patterns of beliefs about HBV transmission routes (Table 2). The two-class solution was retained as optimal. The first class, comprising the vast majority of participants (*n* = 184, 90.2%), was characterized by accurate knowledge of the three major transmission routes (blood, mother-to-child, and sexual) alongside low endorsement of common misconceptions. This class was therefore labeled the “Low-Misconception & High-Knowledge Profile.” The second, smaller class (*n* = 19, 9.8%) demonstrated a contrasting pattern: while also showing high knowledge of correct routes, participants in this group concurrently held a high number of incorrect beliefs about non-transmission routes (e.g., airborne, via sharing meals). This class was labeled the “High-Misconception & High-Knowledge Profile.” The model showed good classification accuracy with a mean posterior probability of 0.995.

**Table 2:** Comparison of participant characteristics by latent class of HBV knowledge and misconceptions.

Characteristic	Overall *N* = 203^1^	High-Misconception & High-Knowledge *N* = 19^1^	Low-Misconception & High-Knowledge *N* = 184^1^	*p*-value^2^
Age group				0.007
≤30 years	98.0 (48.3%)	15.0 (78.9%)	83.0 (45.1%)	
>30 years	105.0 (51.7%)	4.0 (21.1%)	101.0 (54.9%)	
Education level				0.050
High school or below	53.0 (26.1%)	8.0 (42.1%)	45.0 (24.5%)	
College	56.0 (27.6%)	7.0 (36.8%)	49.0 (26.6%)	
Bachelor or above	94.0 (46.3%)	4.0 (21.1%)	90.0 (48.9%)	
Residence				0.3
Rural/village	33.0 (16.3%)	1.0 (5.3%)	32.0 (17.4%)	
Urban/town	170.0 (83.7%)	18.0 (94.7%)	152.0 (82.6%)	
Occupation group				<0.001
Cadre/worker/teacher/farmer/medical staff	60.0 (29.6%)	1.0 (5.3%)	59.0 (32.1%)	
Commercial/housekeeping/unemployed	64.0 (31.5%)	3.0 (15.8%)	61.0 (33.2%)	
Other	79.0 (38.9%)	15.0 (78.9%)	64.0 (34.8%)	
Health insurance				0.3
Self-paid	25.0 (12.3%)	4.0 (21.1%)	21.0 (11.4%)	
Medical insurance	178.0 (87.7%)	15.0 (78.9%)	163.0 (88.6%)	
Timing of HBV diagnosis				0.8
Previously known	111.0 (54.7%)	11.0 (57.9%)	100.0 (54.3%)	
Newly diagnosed	92.0 (45.3%)	8.0 (42.1%)	84.0 (45.7%)	
Aware of liver function results				0.083
Unclear	30.0 (14.8%)	0.0 (0.0%)	30.0 (16.3%)	
Clear	173.0 (85.2%)	19.0 (100.0%)	154.0 (83.7%)	
Aware of family infection status				0.5
Uncertain	113.0 (55.7%)	9.0 (47.4%)	104.0 (56.5%)	
Certain	90.0 (44.3%)	10.0 (52.6%)	80.0 (43.5%)	
Info source: hospital/doctors	183.0 (90.1%)	19.0 (100.0%)	164.0 (89.1%)	0.2
Info source: internet search	174.0 (85.7%)	12.0 (63.2%)	162.0 (88.0%)	0.009
Info source: social media	166.0 (81.8%)	15.0 (78.9%)	151.0 (82.1%)	0.8
Info source: friends/colleagues	24.0 (11.8%)	4.0 (21.1%)	20.0 (10.9%)	0.3
Info source: books/magazines	17.0 (8.4%)	2.0 (10.5%)	15.0 (8.2%)	0.7
Info source: NGOs	8.0 (3.9%)	1.0 (5.3%)	7.0 (3.8%)	0.6
Info source: community lectures	32.0 (15.8%)	5.0 (26.3%)	27.0 (14.7%)	0.2
Perceived infectivity				0.3
Not/low infectious	16.0 (7.9%)	0.0 (0.0%)	16.0 (8.7%)	
Moderate	38.0 (18.7%)	2.0 (10.5%)	36.0 (19.6%)	
High	56.0 (27.6%)	8.0 (42.1%)	48.0 (26.1%)	
Very high	93.0 (45.8%)	9.0 (47.4%)	84.0 (45.7%)	
Perceived disease severity				0.4
Harmless	16.0 (7.9%)	0.0 (0.0%)	16.0 (8.7%)	
Mild/Not severe	56.0 (27.6%)	4.0 (21.1%)	52.0 (28.3%)	
Severe	41.0 (20.2%)	6.0 (31.6%)	35.0 (19.0%)	
Very severe	90.0 (44.3%)	9.0 (47.4%)	81.0 (44.0%)	
Handwashing habit (before meals/after toilet)	181.0 (89.2%)	8.0 (42.1%)	173.0 (94.0%)	<0.001
Knowledge score (0–3)				<0.001
0	1.0 (0.5%)	0.0 (0.0%)	1.0 (0.5%)	
1	59.0 (29.1%)	0.0 (0.0%)	59.0 (32.1%)	
2	78.0 (38.4%)	3.0 (15.8%)	75.0 (40.8%)	
3	65.0 (32.0%)	16.0 (84.2%)	49.0 (26.6%)	
Misconception score (0–6)				<0.001
1	3.0 (1.5%)	3.0 (15.8%)	0.0 (0.0%)	
2	9.0 (4.4%)	7.0 (36.8%)	2.0 (1.1%)	
3	17.0 (8.4%)	5.0 (26.3%)	12.0 (6.5%)	
4	110.0 (54.2%)	4.0 (21.1%)	106.0 (57.6%)	
5	55.0 (27.1%)	0.0 (0.0%)	55.0 (29.9%)	
6	9.0 (4.4%)	0.0 (0.0%)	9.0 (4.9%)	

^1^*n* (%).

^2^Fisher’s exact test; Fisher’s Exact Test for count data with simulated *p*-value (based on 10,000 replicates).

*p*-values are derived from Fisher’s exact test. For the Knowledge Score, the significant *p*-value reflects a difference in the distribution of scores between groups, not a difference in the median level, critically, both groups achieved the same median Knowledge Score of 3 (maximum), demonstrating that the High-Misconception group is not characterized by low factual knowledge but rather by the coexistence of correct knowledge with persistent misconceptions. Additionally, 100% of participants in the High-Misconception group reported being clear about their liver function results, further supporting their characterization as health-conscious individuals. A higher Misconception Score indicates endorsement of more misconceptions.

### Validation of the latent classes

To descriptively characterize the two latent classes in a clinically interpretable manner, we compared them on two summary scores derived from the same set of knowledge items but constructed using a different coding scheme.

**Figure 1:**
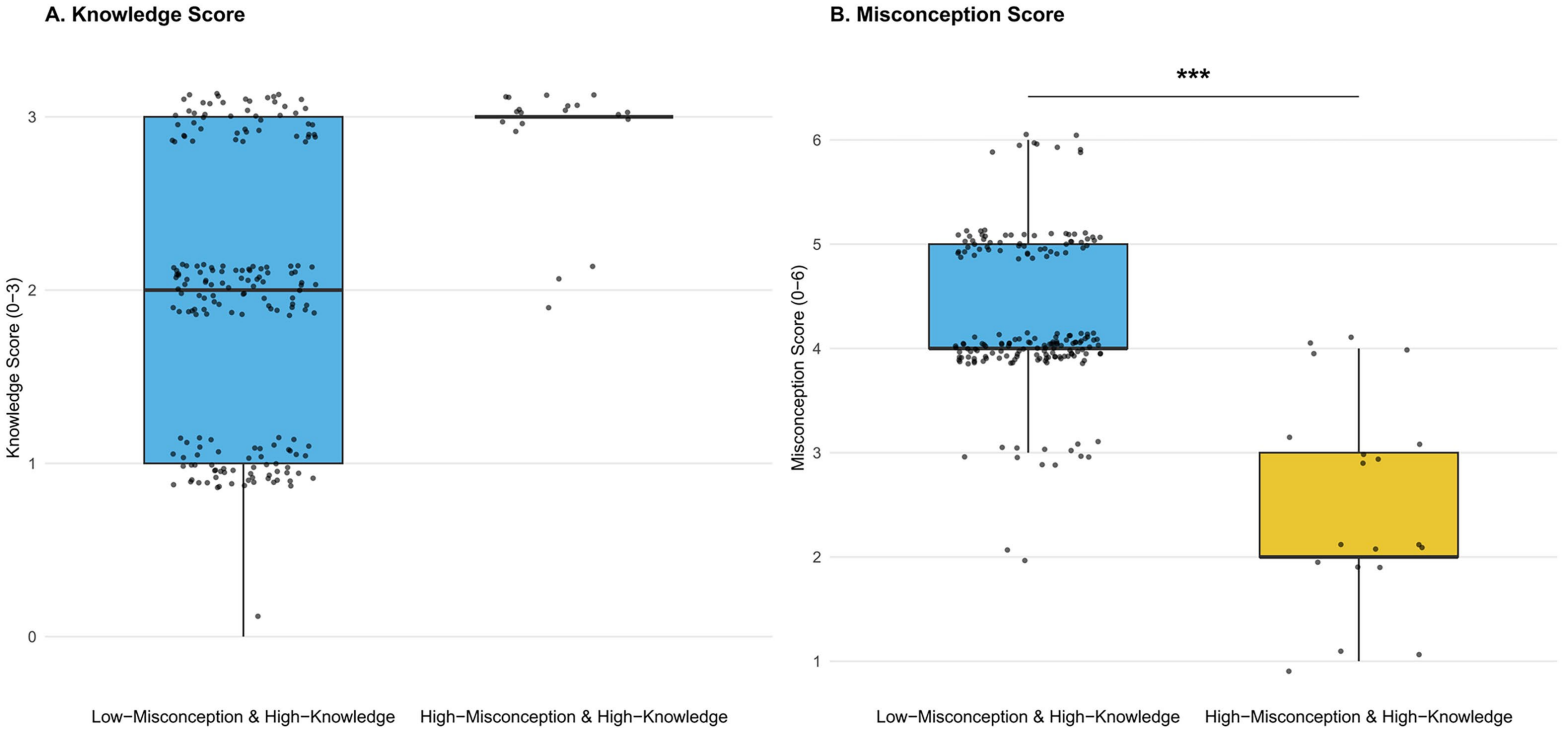
Descriptive characterization of latent classes: divergence in Misconception scores. Comparison of **(A)** Knowledge Score (range 0–3) and **(B)** Misconception score (range 0–6) between the Low-Misconception & High-Knowledge Profile (LM-HK) and the High-Misconception & High-Knowledge Profile (HM-HK). In the box plots, the center line represents the median, the box limits indicate the interquartile range (IQR), and points show individual data points. *p* < 0.001 for the comparison in Panel **(B)** (Wilcoxon rank-sum test), confirming that the HM-HK profile harbors significantly more misconceptions than the LM-HK profile.

While both groups had equally high Knowledge Scores (correct transmission routes, range 0–3), their Misconception Scores (incorrect routes, range 0–6) differed drastically. Validation confirmed a drastically higher Misconception Score in the high-misconception group (median = 3, IQR: 2–4) compared to the low-misconception group (median = 0, IQR: 0–1) (*p* < 0.001, Wilcoxon rank-sum test). Both groups achieved the same median Knowledge Score (3, the maximum possible). The statistically significant difference in the distribution of Knowledge Scores (*p* < 0.001, Wilcoxon rank-sum test) reflects differences in response patterns rather than a meaningful difference in factual knowledge; importantly, the HM-HK profile is not characterized by lower knowledge but by the coexistence of accurate knowledge with a high burden of misconceptions (Table 2; Figure 1).

### Characteristics associated with knowledge profiles

A comprehensive comparison of sociodemographic, clinical, and perceptual characteristics between the two profiles is presented in Table 2. Significant differences were observed in several key variables. Participants in the High-Misconception & High-Knowledge Profile were significantly younger, with 78.9% aged ≤30 years compared to 45.1% in the low-misconception group (*p* = 0.007). The distribution of education levels also differed between groups (*p* = 0.050), with a higher proportion of participants in the High-Misconception & High-Knowledge Profile having a high school education or below (42.1% vs. 24.5%). A striking difference was found in occupation (*p* < 0.001), where 78.9% of the high-misconception group fell into the “Other” category, compared to 34.8% in the low-misconception group. Regarding information sources, the high-misconception group was significantly less likely to report using the internet (63.2% vs. 88.0%, *p* = 0.009). No significant differences were found between the groups in perceived infectivity (*p* = 0.3), perceived disease severity (*p* = 0.4), or most clinical features. Notably, 100% of participants in the high-misconception group reported being ‘clear’ about their liver function results, compared to 83.7% in the low-misconception group (*p* = 0.083). While not statistically significant, this pattern further supports the characterization of the cognitive conflict profile as highly health-conscious individuals who actively monitor their clinical status, rather than knowledge-deficient individuals (Table 2). An observed behavioral difference was that the high-misconception group reported a significantly lower prevalence of handwashing habits (42.1% vs. 94.0%, *p* < 0.001). As noted in the discussion, because handwashing is not a specific preventive measure against HBV transmission, this finding should not be interpreted as a direct failure of HBV prevention. Rather, it may reflect a broader disjunction between holding numerous misconceptions and engaging in general hygiene practices, a pattern that merits further investigation but whose interpretation is limited by the cross-sectional design and lack of behavioral mechanism data.

### Factors associated with the High-Misconception & High-Knowledge Profile

Due to the small size of the HM-HK profile (*n* = 19), multivariable regression was not feasible. As an exploratory analysis, we performed univariable logistic regression to generate hypotheses regarding factors potentially associated with belonging to this profile (Table 3). These results should be interpreted as hypothesis-generating and require validation in larger samples. Education level, treated as an ordinal variable, was a significant protective factor. With each increasing level of education, the odds of belonging to the cognitive conflict profile decreased by approximately 48% (Crude Odds Ratio [OR] = 0.52, 95% Confidence Interval [CI]: 0.29–0.92, *p* = 0.027).

**Table 3:** Univariate logistic regression for factors associated with the cognitive conflict profile (high-misconception & concurrent high-knowledge).

Characteristic	Crude OR	95% CI	*p*-value
Education level (per one-level increase)	0.52	0.29, 0.92	0.027

CI = Confidence interval, OR = Odds ratio.

## Discussion

This study applied latent class analysis (LCA) to move beyond the conventional description of average knowledge levels and identified two distinct cognitive profiles regarding HBV transmission among HBsAg-positive pregnant women in China. This study moves beyond documenting the prevalence of misconceptions. By applying LCA, we uncover the internal structure of health cognition in this population. We identified a distinct “High-Misconception & High-Knowledge Profile” (9.8%). Our LCA extends prior work by characterizing the distribution and sociodemographic profile of a high-misconception subgroup within this at-risk population. While the identified profiles differ primarily in the degree of misconception endorsement, their co-occurrence with distinct demographic (younger age, lower education), occupational (“Other” category), and behavioral (lower handwashing, reduced internet use) features supports their clinical and public health relevance. An observed lower handwashing prevalence, while noted, requires cautious interpretation as detailed in the discussion. This suggests that interventions can be designed to specifically target this combination of high misconceptions and their underlying correlates, rather than implying two fundamentally different cognitive processing systems. This profile, coupled with its distinct socio-behavioral features, carries significant implications for refining MTCT prevention strategies.

### The cognitive conflict profile: high misconceptions despite high knowledge

Although the cognitive conflict profile comprised a minority (9.8%, n = 19) of our sample, which is below 10% and notably falls short of the often-cited guideline of 50–100 participants per class for optimal stability, its identification remains clinically and public health relevant. In latent class analysis, a small but distinct class can be reliably estimated when model fit indices are excellent and classification certainty is high. Our selected 2-class model demonstrated the lowest BIC value (1573.62) among all models and an exceptionally high entropy (0.969), indicating clear separation between classes. The high average posterior probability (0.995) further confirms that, given the model, individual classifications are highly certain. However, classification certainty does not guarantee parameter stability for the smaller class; the item-response probabilities for the HM-HK profile (*n* = 19) should be interpreted with caution, and the stability of this class structure requires confirmation in larger samples. However, we acknowledge that the small size of this profile (*n* = 19) limits the statistical power for subgroup analyses, and these findings should be confirmed in larger, more representative samples. More importantly, the existence of this profile serves as a crucial “proof-of-concept” signal. It demonstrates that a significant cognitive risk pattern exists, which could be missed by aggregate-level analyses. The characteristics of this group provide clear, actionable hypotheses for developing targeted screening tools and interventions, which must be validated and refined in larger, future studies.

The identification of this high-misconception subgroup demonstrates that knowledge and misconceptions are not mutually exclusive but can co-occur, representing a severity gradient. While the two profiles might be viewed as a threshold on a continuum of misconception endorsement, the LCA approach empirically identifies the specific point at which the concentration of misconceptions, coupled with distinct demographic and behavioral characteristics, constitutes a clinically meaningful target. Therefore, rather than positing a qualitatively different cognitive structure, we argue that the co-occurrence of known risk factors with high misconception burden creates a compounding vulnerability that is obscured when examining average scores alone (18, 19). This pattern must be interpreted within the specific socio-cultural context of pregnancy in China, a period of heightened health vigilance where women are positioned as primary guardians of fetal well-being. An HBV diagnosis can intensify this pressure, triggering anxiety and a frantic search for information (20, 21).

Our findings are consistent with a digital health literacy gap potentially contributing to this profile. This profile was associated with younger age and a distinct information ecology: while less likely to proactively “search” the internet, they are highly engaged with social media. In the Chinese context, the most prevalent social media platforms include WeChat (which combines messaging with article sharing and group discussions) and Douyin (short-video platform), both of which have become major channels for health information dissemination — and, consequently, misinformation spread, as unverified claims about HBV transmission routes frequently circulate in family groups and algorithmic video feeds (22). This suggests that younger women may be fluent in navigating social platforms but less equipped to critically appraise the health information (and misinformation) that flows within them, making them vulnerable to internalizing prevalent myths that equate HBV with highly contagious everyday threats. Future digital interventions could leverage these same platforms (e.g., hospital-certified WeChat official accounts, Douyin health education channels) to deliver myth-debunking content through trusted sources, thereby meeting this population in their existing information ecosystems.

### A proposed cognitive dissonance framework for understanding the “cognitive conflict” profile

The following interpretation is hypothesis-generating rather than empirically tested in this study. Our study did not directly measure psychological constructs such as anxiety, stigma, or cognitive dissonance, we propose that the observed pattern of co-occurring accurate knowledge and misconceptions can be usefully interpreted through this theoretical lens as a hypothesis for future research. Within a cognitive dissonance framework, “cognitive conflict” could be conceptualized as the psychological discomfort that arises when individuals simultaneously hold inconsistent cognitions, beliefs, or behavioral intentions, and as their tendency to reduce this discomfort by changing behavior, reinterpreting information, adding consonant cognitions, or downplaying the importance of conflicting information (23).

For pregnant women newly diagnosed with hepatitis B, cognitive dissonance may emerge when accurate biomedical knowledge–that hepatitis B is primarily transmitted through blood exposure, sexual contact, and mother-to-child transmission–coexists with persistent misinformation suggesting that the virus can spread through shared meals, kissing, or ordinary daily contact. Existing studies indicate that hepatitis B stigma and misconceptions about transmission are closely associated with exaggerated perceptions of infectiousness (24).

In the context of pregnancy, heightened maternal responsibility, anxiety, and risk sensitivity may further predispose women to accept additional protective behaviors or even overestimate risks as a way of restoring a sense of control. In this sense, the process is better described as the addition of supportive cognitions to alleviate dissonance rather than simply the rejection of scientifically valid knowledge.

Accordingly, effective interventions should go beyond one-way health education and instead integrate emotional support, stigma reduction, and cognitive reframing. Prior literature suggests that interventions addressing hepatitis B stigma require a safe space for expression, explicit correction of misconceptions, and facilitation of attitudinal change, whereas information correction alone is often insufficient to resolve deeply entrenched concerns (25).

The observed dissociation between high perceived severity/infectivity and low handwashing adherence should be interpreted cautiously. Because hepatitis B virus is transmitted primarily through blood and body-fluid exposure, handwashing is not a hepatitis B-specific preventive behavior; therefore, lower handwashing adherence does not necessarily indicate a direct failure of hepatitis B prevention. Rather, the finding may reflect a broader pattern in which misconceptions about transmission coexist with emotional distress, uncertainty, and difficulty translating risk perceptions into appropriate preventive action (26).

In this context, a cognitive dissonance perspective may still be useful. Women who simultaneously hold accurate biomedical knowledge and persistent transmission myths may experience an unstable risk model, which can increase anxiety without necessarily promoting evidence-based action. However, the available literature does not support a definitive claim that such women become behaviorally inactive because of “preventive fatalism” or executive-function depletion. These mechanisms should instead be framed as plausible hypotheses requiring empirical validation (23).

Clinically, this suggests that counseling should focus less on simply encouraging handwashing and more on correcting specific transmission misconceptions, clarifying which preventive measures are actually relevant to HBV, and strengthening confidence in actionable, evidence-based behaviors such as antenatal screening, antiviral prophylaxis when indicated, and newborn immunoprophylaxis (26).

If future research confirms an emotional component to this profile, the constellation of potential anxiety, misconception, and information vulnerability might have consequences beyond biomedical risk. It could foster internalized stigma, unnecessary social isolation, and create barriers to open communication with healthcare providers. However, these specific psychological consequences require direct measurement in future studies. Therefore, addressing this profile requires moving beyond simple knowledge correction to proactively address the emotional and social sequelae of holding conflicting, fear-inducing beliefs during a vulnerable life stage.

### The protective role of education and the primacy of contextual factors

The univariable association between higher educational attainment and a lower likelihood of belonging to the cognitive conflict profile underscores the role of structural health literacy (27, 28). Education often confers not only factual knowledge but also critical thinking skills and the self-efficacy to seek and evaluate information from authoritative sources. However, this finding also hints at underlying social gradients in health. Women with lower educational attainment may face compounded vulnerabilities, including limited access to high-quality health information, less time for health self-management due to socioeconomic pressures, and potentially greater exposure to stigmatizing community narratives about infectious diseases (29). While our study’s digital survey methodology may have under-sampled the most marginalized women (30), the pattern suggests that interventions must be designed with equity in mind, ensuring accessible and low-literacy appropriate materials to bridge this gap.

### Implications for clinical practice and translational pathways

To translate these findings into clinical impact, we propose a multi-level approach integrated into standard antenatal care: (1) Development of Brief Screening Tools: Our LCA item response probabilities can inform the creation of a 2–3 item ultra-brief screener for use during routine antenatal intake. For example, questions probing beliefs about ‘transmission via sharing meals’ or ‘airborne spread’, which showed high discriminatory power in our analysis, could efficiently flag women belonging to the cognitive conflict profile for immediate, targeted counseling (31, 32). (2) Structured Counseling Protocol: For identified women, clinicians should follow a protocol that first empathetically addresses fears and debunks casual transmission myths before reinforcing correct knowledge and MTCT prophylaxis adherence. This can reduce stigma-driven disengagement (33, 34). (3) Prescribing Information: “Prescribe” vetted digital resources (e.g., hospital-certified social media accounts) as adjuvant education, counteracting misinformation (35, 36). (4) Training Healthcare Providers: Equip obstetricians and nurses with skills to deliver this nuanced, patient-centered communication (37, 38). This pathway directly links cognitive profiling to actionable clinical behaviors, aiming to improve maternal mental well-being and, ultimately, MTCT prevention efficacy.

### Strengths and limitations

This study has two major strengths. First, it applied latent class analysis (LCA), a well-established and robust method with particular strengths in uncovering population heterogeneity. Second, the latent classes were systematically characterized and their clinical meaningfulness was demonstrated by comparing them on summary knowledge and misconception scores, thereby enhancing the interpretability of the profiles. The study was based on a large, rigorously collected dataset from a key population targeted for the prevention of mother-to-child transmission, providing a solid and reliable empirical foundation.

Several limitations should also be acknowledged. First, the cross-sectional design precludes causal inference regarding the relationships among information sources, cognitive misconceptions, and class membership. Second, and most pertinent to the LCA findings, the identified high-misconception profile was small (*n* = 19, 9.8%). While the excellent model fit indices (entropy = 0.969, low BIC) support the statistical distinction of this profile, its size is below the commonly recommended threshold of 50–100 participants per class for stable class enumeration. This has two key implications: (1) The precision of the estimated item-response probabilities for this specific class is lower, and the profile’s characteristics should be interpreted as preliminary. (2) As noted, the statistical power for subgroup analyses (e.g., comparisons in Table 2) and particularly for the univariable logistic regression (Table 3) was severely limited. Therefore, the profile’s characteristics and the associated factors (e.g., from the univariable logistic regression) must be considered preliminary and hypothesis-generating, requiring confirmation in larger samples. Any apparent non-significant associations may be attributable to insufficient power rather than true absence of effects. Future translational research should focus on developing and pilot-testing the proposed screening tool and targeted counseling intervention within clinical settings, with subsequent evaluation of their impact on key clinical endpoints, such as maternal anxiety reduction, medication adherence, and MTCT rates. Third, consistent with the parent study, selection bias may be present, as the most socioeconomically disadvantaged pregnant women, particularly those with the lowest educational attainment, may have been underrepresented in the digital survey. Fourth, we acknowledge that the Knowledge and Misconception scores used to characterize the latent classes were derived from the same indicator items used in the LCA model. Therefore, our “validation” should be considered a quasi-external interpretive step. Future studies should validate these profiles against genuine external criteria, such as measures of stigma, anxiety, health-seeking behaviors (e.g., consultation for antiviral therapy), or subsequent adherence to the MTCT prevention cascade. Finally, while this study characterized the types of information sources accessed by participants, it did not systematically assess the quality or specific content of that information, which represents an important direction for future research.

## Conclusion

In conclusion, this LCA-based study reveals that HBsAg-positive pregnant women are not a monolithic group in terms of HBV cognition. We identified a high-misconception subgroup characterized by a high burden of casual-transmission myths alongside good factual knowledge, with distinct sociodemographic and behavioral correlates. The concentration of these features within a small proportion of the sample (9.8%) highlights a specific vulnerability where misconception endorsement intersects with factors such as younger age and reduced internet use. This subgroup represents a potential target for tailored, precision health education interventions. To accelerate progress toward the elimination of HBV MTCT, public health strategies must evolve from universal messaging to culturally competent, precision education. This entails screening for such high-risk cognitive-emotional profiles within antenatal care and delivering targeted, empathetic counseling that not only debunks myths but also addresses underlying anxiety and stigma. Interventions should actively engage with and leverage the digital ecosystems where young pregnant women seek information, while strengthening the supportive, trust-based clinician-patient relationship. Ultimately, empowering HBsAg-positive pregnant women requires building support systems that acknowledge and address the interplay of knowledge, emotion, and social context throughout their pregnancy journey, ensuring that medical advances are fully realized in the lived experience of every woman.

## Data Availability

The raw data supporting the conclusions of this article will be made available by the authors, without undue reservation.
